# A response surface optimization approach to adjust ionic current conductances of cardiac electrophysiological models. Application to the study of potassium level changes

**DOI:** 10.1371/journal.pone.0204411

**Published:** 2018-10-03

**Authors:** Jesús Carro, Esther Pueyo, José F. Rodríguez Matas

**Affiliations:** 1 Universidad San Jorge, Villanueva de Gállego, Zaragoza, Spain; 2 Aragón Institute for Engineering Research, University of Zaragoza, IIS Aragón, Spain; 3 CIBER in Bioengineering, Biomaterials & Nanomedicne (CIBER-BBN), Spain; 4 LaBS, Department of Chemistry, Materials and Chemical Engineering “Giulio Natta”, Politecnico di Milano, Italy; Cinvestav-IPN, MEXICO

## Abstract

Cardiac electrophysiological computational models are often developed from previously published models. The new models may incorporate additional features to adapt the model to a different species or may upgrade a specific ionic formulation based on newly available experimental data. A relevant challenge in the development of a new model is the estimation of certain ionic current conductances that cannot be reliably identified from experiments. A common strategy to estimate those conductances is by means of constrained non-linear least-squares optimization. In this work, a novel methodology is proposed for estimation of ionic current conductances of cardiac electrophysiological models by using a response surface approximation-based constrained optimization with trust region management. Polynomial response surfaces of a number of electrophysiological markers were built using statistical sampling methods. These markers included action potential duration (APD), triangulation, diastolic and systolic intracellular calcium concentration, and time constants of APD rate adaptation. The proposed methodology was applied to update the Carro *et al*. human ventricular action potential model after incorporation of intracellular potassium ([*K*^+^]_*i*_) dynamics. While the Carro *et al*. model was well suited for investigation of arrhythmogenesis, it did not allow simulation of [*K*^+^]_*i*_ changes. With the methodology proposed in this study, the updated Carro *et al*. human ventricular model could be used to simulate [*K*^+^]_*i*_ changes in response to varying extracellular potassium ([*K*^+^]_*o*_) levels. Additionally, it rendered values of evaluated electrophysiological markers within physiologically plausible ranges. The optimal values of ionic current conductances in the updated model were found in a notably shorter time than with previously proposed methodologies. As a conclusion, the response surface optimization-based approach proposed in this study allows estimating ionic current conductances of cardiac electrophysiological computational models while guaranteeing replication of key electrophysiological features and with an important reduction in computational cost with respect to previously published approaches. The updated Carro *et al*. model developed in this study is thus suitable for the investigation of arrhythmic risk-related conditions, including those involving large changes in potassium concentration.

## 1 Introduction

The last decades have seen an extraordinary development of cardiac computational electrophysiological models. The possibilities they offer to investigate the electrical behavior of the heart together with the large increase in computing capability have boosted such development. This has been accompanied in many instances by an increase in model complexity, even if the main principles and structure of the first published models remain unaltered.

An action potential (AP) model is described by a system of coupled differential equations that involve the transmembrane current flow as well as dynamic changes in intracellular ionic concentrations. For each ionic current a mathematical formulation is employed to represent characteristics like gating or permeability of the corresponding ion channels. Those formulations rely on a number of model parameters which values are commonly estimated based on experimental data obtained from specific protocols for each ionic current. In this context parameter estimation presents various shortcomings intrinsic to the voltage-clamp experimental protocols [1]: i) insufficient separation of ionic current activation and inactivation processes; ii) use of non-selective pharmacological ion channel blockers; and iii) measurement of non-physiological solutions.

Despite these limitations, most parameter values of individual ionic currents have been identified in the past using independent sets of experiments. However, parameters describing ionic current conductances have not always been successfully characterized from experimental datasets, in large part due to the sensitivity of some ion channels to the cell isolation procedure performed before application of the voltage-clamp protocols [2]. Also, experimental studies may have purposely selected cells with large ionic currents, which interfere with model development based on such data [3]. For those reasons, a common procedure to identify individual ionic current conductances is based on model parameter fitting aimed at reproducing plausible values of electrophysiological AP properties like AP duration (APD), triangulation or upstroke velocity [4, 5]. Other models include also constraints based on experimental data measured after blocking certain ionic currents [6], imposed to obtain a correct input resistance [3], or based on additional properties like APD rate adaptation and rate dependence of ionic concentrations [7].

Cardiac electrophysiological computational models are commonly used to analyze the effect of drugs or to study the mechanisms underlying pathological conditions. Under those circumstances, the correct characterization of ionic current conductances is crucial. Overestimation or underestimation of current conductance values could lead to completely erroneous predictions. For this reason, while for many years the adjustment of current conductances in cardiac computational models has been frequently performed manually [8], a number of techniques have been developed in recent years to help in the process of parameter identification. Gradient descent methods [9, 10] and genetic algorithms [11–14] have been used for this purpose in several studies. Gradient descent methods rely on computing local gradients of the error function to determine a search direction towards a minimum. On the other hand, genetic algorithms use ideas from evolutionary biology and test different combinations (populations) of parameters to optimize the error function. In each iteration they create a new population based on the best results from previous iterations.

Gradient descent methods and genetic algorithms have a small degree of parallelization and, thus, other techniques with higher parallelization capability could better exploit the possibilities of current computing infrastructures. In this work a novel methodology is proposed for identification of parameters describing ionic current conductances of cardiac electrophysiological models by using an optimization algorithm with trust region management [15]. The algorithm minimizes an objective function subject to non-linear constraints associated with a number of electrophysiological properties. The objective function and constraints are approximated by polynomial response surfaces which allow the optimization algorithm searching for a solution to the problem without performing a direct evaluation of the model. The use of a response surface helps to reduce the number of iterations of the algorithm and, thus, reduce the time cost of optimizing the model. To guarantee the ability of the model to represent physiological behavior, the region where the solution is searched for is limited based on experimentally reported values of electrophysiological properties like APD, AP triangulation, diastolic and systolic intracellular calcium concentrations and time constants of APD rate adaptation to abrupt changes in cycle length (CL). The proposed methodology is applied to adjust the ionic conductances of the human ventricular *Carro-Rodriguez-Laguna-Pueyo* (CRLP) model [7] after incorporation of intracellular potassium, [*K*^+^]_*i*_, dynamics. The modified CRLP model is compared against the most commonly used human ventricular AP models, namely the ten Tusscher-Panfilov 2006 (TP06) [16], Grandi-Pasqualini-Bers (GPB) [4], O’Hara-Rudy (ORd) [17] models as well as with the Carro-Rodriguez-Laguna-Pueyo (CRLP) [7] model.

The relevance of accounting for [*K*^+^]_*i*_ dynamics is demonstrated by simulating a number of transitions in the extracellular potassium concentration, as in hemodialysis studies [18], and comparing the behavior of the original and modified CRLP models.

## 2 Materials and methods

### 2.1 Optimization problem

#### 2.1.1 General formulation

A minimization problem with constraints can be written as:

$$\begin{array}{ccc} & & \text{min}\;f(x) \end{array}$$ (1)

subject to

$$\begin{array}{ccc} & & h(x)=0, \end{array}$$ (2)

$$\begin{array}{ccc} & & g(x)\geq 0, \end{array}$$ (3)

$$\begin{array}{ccc} & & x^{L}\leq x\leq x^{U}, \end{array}$$ (4)

where **x** is the vector of decision variables, **x**^*L*^ and **x**^*U*^ their respective lower and upper bounds, **f**(**x**) is the objective function, and **h**(**x**) and **g**(**x**) are vector functions of equality and inequality constraints, respectively.

#### 2.1.2 Particularization to ionic conductances estimation

The general minimization problem described in previous section was particularized in this study for estimation of ionic current conductances. Since different ionic current conductances *G*_*i*_ may differ by orders of magnitude, the following scaling was adopted for each of the elements of vector **x**:

$$\begin{array}{c} x_{i}=20\cdot log_{10}\frac{G_{i}}{G_{i}^{0}}, \end{array}$$ (5)

where *G*_*i*_ is the unscaled ionic current conductance and $G_{i}^{0}$ is its initial value in the model.

The objective function is defined for each particular problem. In this study, it was represented by the integral of the total potassium current during a cardiac cycle at 1 Hz steady-state pacing, as fully described in 2.2.3. No equality constraint function was defined in this case. The inequality constrain function was defined by imposing a number of electrophysiological properties to remain within experimentally observed ranges:

$$\begin{array}{c} m_{LL}\leq m\left(x\right)\leq m_{UU} \end{array}$$ (6)

where **m**_*LL*_ and **m**_*UU*_ are the lower and upper bounds delimiting physiological ranges, respectively, and **m**(**x**) is the vector of electrophysiological properties calculated for **x**.

Eq 6 was reformulated to be consistent with the general formulation of the inequality constraints described in Eq 3:

$$\begin{array}{c} g_{j}\left(x\right)=|\frac{m_{j}\left(x\right)-m_{j,CR}}{m_{j,RR}}|-1\leq 0, \end{array}$$ (7)

where mj,CR=(mj,UL+mj,LL)2 is the central point of the experimental range for the electrophysiological property *j* and mj,RR=(mj,UL−mj,LL)2 is the distance between *m*_*j*,*CR*_ and each of the limits of the experimental range.

#### 2.1.3 Optimization using response surfaces

The optimization algorithm used in this study seeks for a solution of the minimization problem, Eqs 1–4, by solving a sequence of approximate constrained optimization problems within a region of trust. For the iteration *k* of the algorithm, the approximate problem reads as:

$$\begin{array}{ccc} & & \text{min}\;\tilde{f}\left(x\right) \end{array}$$ (8)

subject to

$$\begin{array}{ccc} & & \tilde{h}\left(x\right)=0, \end{array}$$ (9)

$$\begin{array}{ccc} & & \tilde{g}\left(x\right)\geq 0, \end{array}$$ (10)

$$\begin{array}{ccc} & & ∥x∥\leq \Delta^{k}, \end{array}$$ (11)

where $\tilde{f}\left(x\right)$, $\tilde{h}\left(x\right)$ and $\tilde{g}\left(x\right)$ are response surface approximations (RSA) of *f*(**x**), **h**(**x**), and **g**(**x**) around **x**^*k*^, respectively, and Δ^*k*^ is the corresponding trust region radius. The trust region radius defines a region of trust in which the RSA is assumed to be reliable. The validity of the RSA can be evaluated through the trust region ratio test. For the objective function *f*(**x**), the trust region ratio for iteration *k* was defined as:

$$\begin{array}{c} \rho_{0}^{k}=\frac{f\left(x^{k}\right)-f\left(x^{k,*}\right)}{\tilde{f}\left(x^{k}\right)-\tilde{f}\left(x^{k,*}\right)}, \end{array}$$ (12)

where *x*^*k*,^* is a solution of the minimization problem given by Eqs 8–11. This definition of the trust region ratio is based on the consideration that $\rho_{0}^{k}\to 1$ as the trust region radius Δ^*k*^ → 0. If $\rho_{0}^{k}\leq 0$, the approximation is deemed as unacceptable and, consequently, the trust region radius is reduced. If $\rho_{0}^{k}\approx 1$ and ‖**x**^*k*^,* − **x**^*k*^‖ = Δ^*k*^, the RSA is evaluated as acceptable and Δ^*k*^ is increased. If $0<\rho_{0}^{k}<1$, Δ^*k*^ is kept unchanged, but the RSA is rebuilt around *x*^*k*,^*. Note that $\rho_{0}^{k}>>1$ is also indicative of poor RSA and Δ^*k*^ is reduced, but the optimizer is considered to be moving in the right direction. The update of the trust region radius offers significant flexibility depending on the implementation. A particular updating rule will be presented in the following section.

The algorithm proposed in this work is based on the one described in [15] but it simplifies some aspects on how to manage and use the response surfaces and it reduces the number of times the database needs to be generated. Instead of considering the augmented Lagrangian of the problem, the proposal of this study is based on generating the response surface of the model and looking for the minimum in this surface. Subsequently, this solution is validated by using the trust region test in both the minimization function and the constraints of the model. This notably reduces the computational cost, as the database is generated once per iteration rather than every time there is a change in the parameters of the augmented Lagrangian.

#### 2.1.4 Database generation and response surface approximation

One of the key elements of the minimization algorithm is building the RSA of the objective and constraint functions that will be used to solve the approximate minimization problem. To construct a response surface for a function of *n* variables, a large number of sample points are required. Also, to construct a reliable RSA, the sample points must be adequately distributed. Sampling techniques based on design of experiments are commonly used for this purpose [19]. In this study, full factorial designs, which require 3^*n*^ sampling points, were considered. A full factorial design consists of a three-level factorial: the corner points of a hypercube, plus the center of the hypercube and of each facet, plus the midpoints of the lines connecting the corners of the hypercube. In the next sections, the set of results of the sampling technique in an iteration of the algorithm for both the minimization and constraint functions will be referred to as *database*. Second order polynomial response surfaces were used in all cases.

#### 2.1.5 Minimization algorithm

**Fig 1:**
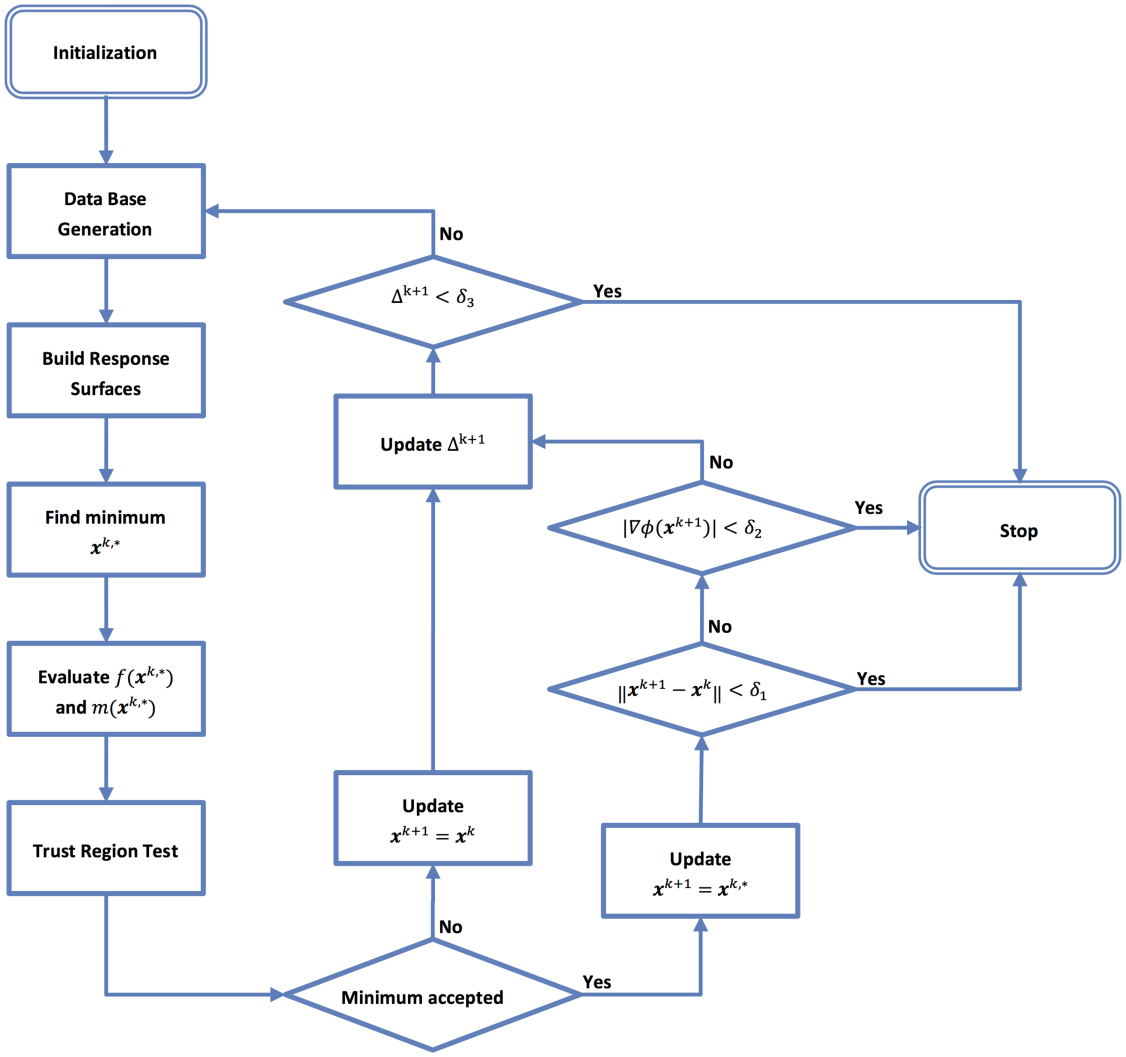
Flow chart of the proposed algorithm.

The minimization algorithm implemented in this work is shown in Fig 1. The database was computed only once per iteration of the algorithm or whenever the solution was rejected and the trust region radius was reduced. The trust region ratio was used to validate the RSA for the objective function and constraints independently.

The basic steps of the algorithm shown in Fig 1 are briefly described in the following. The index *k* indicates the iteration of the algorithm, *i* indicates the variable index and *j* represents the constraint index.

#### Step 0: Initialization

Define values for the tolerances *δ*_1_ (solution tolerance), *δ*_2_ (gradient tolerance) and *δ*_3_ (improvement tolerance), the minimum acceptable value *ρ*_*L*_ for the trust region ratio to consider a trust region as acceptable and an upper limit Δ^max^ for the trust region radius. Define an initial value Δ^0^ for the size of the trust region and a starting point **x**^0^ for the variables of the problem. Set the iteration counter at *k* = 0.

#### Step 1: Database generation

Compute the database as described in section 2.1.4.

#### Step 2: Response surfaces

Build the RSA of the objective function and constraints of the problem around the current iterate **x**^*k*^.

#### Step 3: Find the minimum of the approximate problem

Find the minimum **x**^*k*,^* of the constrained minimization problem defined by Eqs 4–11 in the trust region limited by Δ^*k*^.

#### Step 4: Trust region test

Evaluate *f* and each of the properties *m*_*i*_ in **x**^*k*,^* and calculate the trust region ratio for the objective and constraint functions as in Eq 12.

$$\begin{array}{ccc} \rho_{0}^{k} & = & \frac{f\left(x^{k,*}\right)-f\left(x^{k}\right)}{\tilde{f}\left(x^{k,*}\right)-\tilde{f}\left(x^{k}\right)} \end{array}$$ (13)

$$\begin{array}{ccc} \rho_{j}^{k} & = & \frac{m_{j}\left(x^{k,*}\right)-m_{j}\left(x^{k}\right)}{{\tilde{m}}_{j}\left(x^{k,*}\right)-{\tilde{m}}_{j}\left(x^{k}\right)},\;1\leq j\leq M. \end{array}$$ (14)

The solution **x**^*k*,^* was accepted when $\rho_{0}^{k}>0$ and one of the following two criteria was met: i) the constraints in **g**(**x**) were feasible or; ii) $|\rho_{j}^{k}-1|\leq \rho_{L},\forall j$. For this work, *ρ*_*L*_ = 0.25 was used. If the solution was accepted, the flag $flg_{TR}^{k}=true$ was set and **x**^*k*+1^ ≔ **x**^*k*,^*, otherwise $flg_{TR}^{k}=false$ and **x**^*k*+1^ ≔ **x**^*k*^.

#### Step 5: Trust region radius update

The trust region radius was updated as follows:

$$\begin{array}{c} \Delta^{k+1}=\{\begin{array}{ccc} 0.25\Delta^{k} & \text{if} & flg_{TR}^{k}=false,\\ \Delta^{k} & \text{if} & flg_{TR}^{k}=true\;\;\text{and}\;\;∥x^{k,*}-x^{k}∥<\Delta^{k},\\ 2\Delta^{k} & \text{if} & flg_{TR}^{k}=true\;\;\text{and}\;\;∥x^{k,*}-x^{k}∥=\Delta^{k}. \end{array} \end{array}$$ (15)

#### Step 6: Algorithm termination

The algorithm ended if one of the following conditions was satisfied:

1. ‖**x**^*k*+1^ − **x**^*k*^‖ < *δ*_1_ and $flg_{TR}^{k}=true$: The distance between the last two accepted iterates was too small to be significant.
2. ‖**∇***ϕ*(**x**^*k*+1^)| < *δ*_2_ and $flg_{TR}^{k}=true$: The gradient of the Lagrangian function was too small. The Lagrangian function was defined as:
  $$\begin{array}{c} \phi \left(x\right)=f\left(x\right)+\sum_{j=1}^{M}\lambda_{j}^{k}\cdot m_{j}\left(x\right) \end{array}$$ (16)
  where $\lambda_{j}^{k}$ are the Lagrange multipliers obtained in the constrained minimization problem defined by Eqs 4–11 in the trust region (limited by Δ^*k*^) in Step 3.
3. Δ^*k*+1^ < *δ*_3_: The trust region radius became too small.

If none of these criteria was satisfied, then set *k* ≔ *k* + 1 and go to step 1.

### 2.2 Upgrade of the CRLP model

#### 2.2.1 Human ventricular AP models

Several computational models have been proposed in the last ten to fifteen years to represent the electrical behavior of human ventricular cells. For some years, the *tenTusscher-Panfilov* model (TP06) [16] was the most commonly used human ventricular model for electrophysiological investigations. In 2010, the *Grandi-Pasqualini-Bers* (GPB) model [4] was developed based on the Shannon *et al*. [20] rabbit ventricular model. The GPB model provided a detailed description of intracellular calcium dynamics and calcium buffers that allowed more accurate representation of certain experimental behaviors than the TP06 model [7]. The *O’Hara-Rudy dynamic* (ORd) model was proposed in 2011 [17] based on more extensive experimental data from undiseased human ventricles. In the same year, a modification of the GPB model was proposed as the *Carro-Rodriguez-Laguna-Pueyo* (CRLP) model [7]. This modification improved the GPB model response to allow replication of various experimentally-measured risk-related markers, thus rendering it more suitable for investigation of cardiac arrhythmias. In 2015, Himeno *et al*. proposed a new human ventricular cell model [21]. As opposed to the previously described models, the Himeno et al. model included a description of excitation-contraction. The model comparison performed in the present study was restricted to purely electrophysiological models like the TP06, GPB, ORd and CRLP models.

One of the differences between the CRLP model and the ORd and TP06 models lies in the representation of [*K*^+^]_*i*_ dynamics. In the CRLP model, as in the GPB model, [*K*^+^]_*i*_ was assumed to be constant. Under steady-state conditions, [*K*^+^]_*i*_ variations during an AP can be neglected. Nevertheless, when there is a change in the investigated conditions or if the model is somewhat altered, [*K*^+^]_*i*_ may reach a new steady-state value. This is the case, for instance, when the stimulation frequency is varied, as [*K*^+^]_*i*_ becomes reduced in response to increased stimulation frequency [17, 22]. Another scenario where [*K*^+^]_*i*_ dynamics are relevant is in the simulation of acute ischemia. Dutta et al. showed the limitations of using the GPB model with constant [*K*^+^]_*i*_ [23]. In the present work, the methodology described in section 2.1 was used to incorporate [*K*^+^]_*i*_ dynamics into the CRLP model while retaining its capacity to reproduce experimentally observed values of arrhythmic risk-related markers.

#### 2.2.2 [*K*^+^]_*i*_ dynamics in the CRLP model

[*K*^+^]_*i*_ dynamics were introduced through the following equation:

$$\begin{array}{c} \frac{d}{dt}{\left[K^{+}\right]}_{i}=-I_{K,tot}\cdot \frac{C_{mem}}{V_{myo}\cdot F_{rdy}} \end{array}$$ (17)

where $I_{K_{tot}}$ is the total potassium current, *C*_*mem*_ is the membrane specific capacitance, *V*_*myo*_ is the bulk cytosol volume and *F*_*rdy*_ is Faraday’s constant.

$I_{K_{tot}}$ was calculated as the sum of all potassium currents and the stimulus current:

$$\begin{array}{c} I_{K_{tot}}=I_{to}+I_{Kr}+I_{Ks}+I_{K1}-2\cdot I_{Na,K}+I_{{Ca}_{K}}+I_{Kp}+I_{stim} \end{array}$$ (18)

where *I*_*to*_ is the *transient outward* potassium current, *I_Kr_* is the *rapidly activating* potassium current, *I_Ks_* is the *slowly activating* potassium current, *I_K1_* is the *inward rectifier* potassium current, *I_Na,K_* is the *sodium-potassium pump* current, $I_{{Ca}_{K}}$ is the current generate by potassium ions passing through the *L-type calcium channels*, *I_Kp_* is the *plateau* potassium current, and *I*_*stim*_ is the stimulus current.

**Fig 2:**
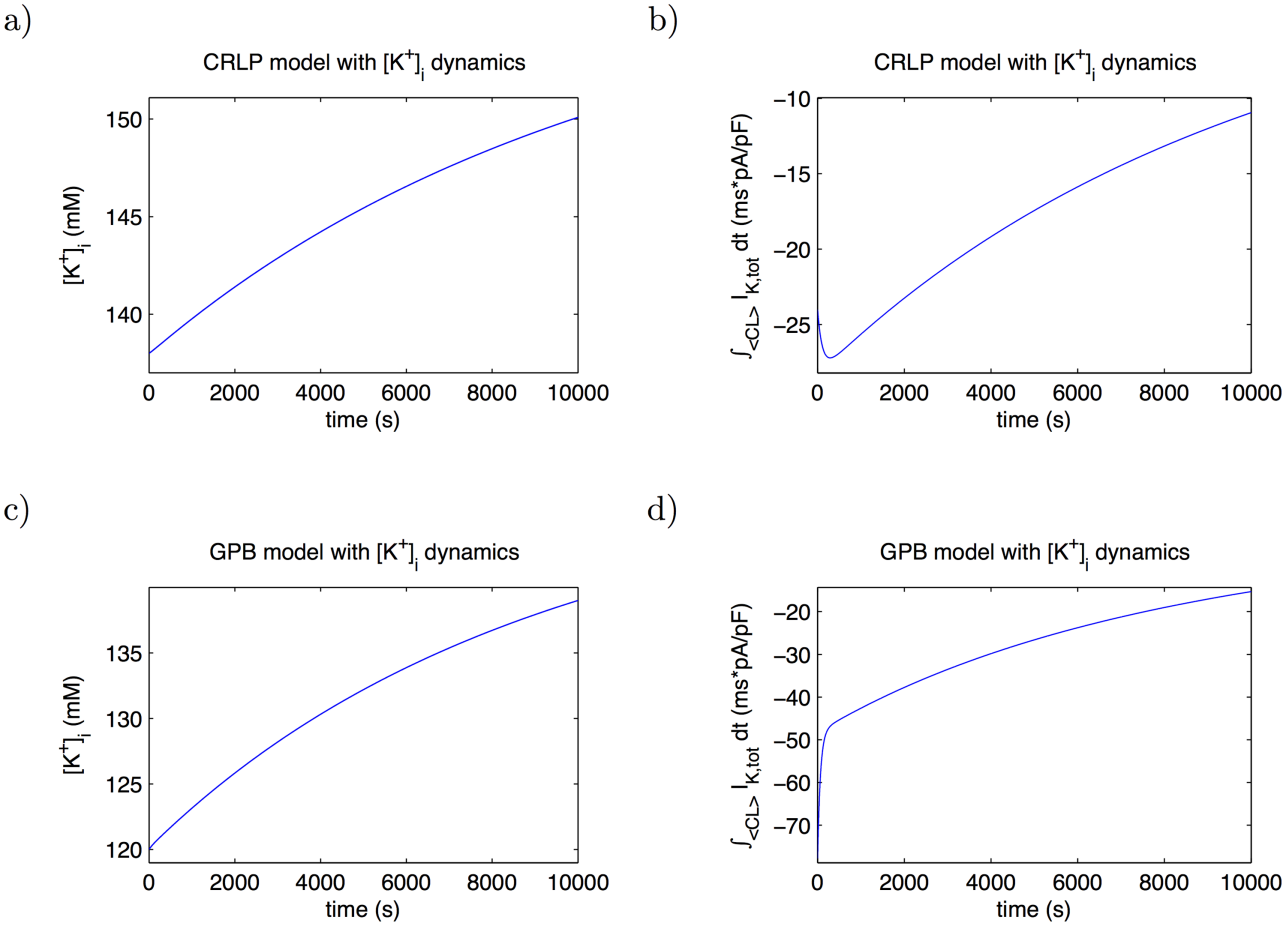
Temporal evolution of [*K*^+^]_*i*_ in CRLP and GPB epicardiacl cell models after introducing [*K*^+^]_*i*_ dynamics. a) and c) show [*K*^+^]_*i*_ evolution when the models (CRLP and GPB, respectively) are stimulated with a CL of 1000 ms. b) and d) show the evolution of the integral of *I*_*K*,*tot*_ during one cycle for the same simulation as in a) and c).

If Eq 17 is directly added to the CRLP and GPB models, [*K*^+^]_*i*_ drifts (Fig 2, panels a) and c), respectively). The continuous increase in [*K*^+^]_*i*_ is due to a larger potassium influx in each cardiac beat, which can in turn be explained by an imbalance in the potassium current. Although it is not straightforward to establish a physiological range for [*K*^+^]_*i*_ because there are no experimental measurements of this concentration, all human ventricular cell models published in the literature, despite having different [*K*^+^]_*i*_ values under steady-state conditions, they all lie within the range 120-145 mM. This range is in agreement with the corresponding experimentally reported values for the resting membrane potential, which is close to the Nernst potential for potassium [17].

In Fig 2, panels b) and d), the integral of the total potassium current during one cardiac cycle is shown, which gives an idea of how far the models are from equilibrium. The above noted issue associated with the introduction of [*K*^+^]_*i*_ dynamics into the CRLP model can be sorted out by readjusting the current conductances to maintain [*K*^+^]_*i*_ homeostasis. Since, at the same time, the performance of the model in terms of representation of electrophysiological behavior wants to be retained, a constrained optimization problem was formulated in this study. This problem was tackled by applying the response surface-based optimization methods presented in 2.1.

For further model assessment, optimization was performed as described in the next paragraph while considering different initial [*K*^+^]_*i*_ values in the range 120-145 mM.

#### 2.2.3 Optimization-driven model parameter update

A new set of current conductances for the modified CRLP model was sought such that potassium homeostasis was preserved while a number of electrophysiological properties were guaranteed to remain within experimental limits. This was stated as a constrained optimization problem of the form:

$$\begin{array}{c} \text{min}\;{\left(\int_{<CL>}I_{K,tot}\left(x\right)\right)}^{2} \end{array}$$ (19)

subject to

$$\begin{array}{ccc} & & m_{LL}\leq m\left(x\right)\leq m_{UU}, \end{array}$$ (20)

$$\begin{array}{ccc} & & x^{L}\leq x\leq x^{U}, \end{array}$$ (21)

where **x** is the vector of scaled current conductances (see 2.1.2), with lower an upper bounds **x**^*L*^ and **x**^*U*^, respectively, ∫_<*CL*>_ represents the integral during a cardiac cycle and **m** is a vector function of selected electrophysiological properties with lower and upper physiological bounds defined by vectors **m**_*LL*_ and **m**_*UU*_. Initialization was defined based on the epicardial CRLP model (where [*K*^+^]_*i*_ takes a constant value of 138 mM). Other initializations were additionally tested, as described in 2.2.2.

#### 2.2.4 Constraint functions

Seven ventricular electrophysiological properties of epicardial cells were considered for definition of inequality constraints in 2.2.3:

- **APD_90_**: APD is considered the main preclinical marker of drug cardiotoxicity. APD prolongation has been linked to long QT syndrome and increased risk of developing Torsades de Pointes [24, 25]. In this study, APD_90_ was used to denote AP duration at 90% repolarization.
- **Triangulation**: This marker quantifies the shape of the final part of the AP and is defined as the difference between APD at 90% and 50% of repolarization. Low triangulation values indicate square APs, while high values indicate triangular APs. Triangulation has been proposed as a marker of pro-arrhythmia [24], with long APD_90_ values without triangular APs considered as anti-arrhythmic and with triangular APs considered as pro-arrhythmic in general.
- **Systolic and diastolic intracellular calcium ([*Ca*^2+^]_*i*_) levels**: Calcium transient properties evaluated at different pacing frequencies have been reported as arrhythmic risk markers [26, 27]. In this study, diastolic (resting level) and systolic (peak value) calcium transient levels were evaluated at 0.5 Hz and 1 Hz steady-state pacing.
- **Time constants of APD_90_ adaptation to abrupt changes in CL**: Adaptation of ventricular repolarization duration to abrupt changes in CL has been proposed as an arrhythmic risk marker [28]. In this study, the dynamics of APD_90_ adaptation to abrupt changes in CL were fitted by two exponentials with associated time constants *τ*_*fast*_ and *τ*_*slow*_, following the methodology proposed in Pueyo *et al*. [29]. Only *τ*_*slow*_ has been considered since there is no available quantitative experimental data on *τ*_*fast*_ values.

Two stimulation protocols were used to calculate the above described markers:

- **Steady-state protocol**: A train of 3,000 stimulation pulses was delivered at a given CL. Simulations were carried out using two different CL values: 1,000 and 2,000 ms. The evaluated markers were: APD_90_ and triangulation at 1,000 ms; and diastolic and systolic [*Ca*^2+^]_*i*_ at 1,000 and 2,000 ms.
- **Abrupt changes in CL protocol**: The cell was stimulated with a CL of 1,000 ms for 8 minutes and then with a CL of 600 ms for additional 8 minutes. APD_90_ dynamics after the abrupt CL change were best fitted by two exponentials, the second of which was characterized by *τ*_*slow*_.

#### 2.2.5 Physiological bounds for constraint functions

Lower and upper bounds **m**_*LL*_ and **m**_*UU*_ were defined according to physiological experimental values reported in the literature for the analyzed properties. The selected range for APD_90_ was 280-310 ms. Even though some studies have reported wider ranges, up to 271-366 ms [30–32], a restricted interval was used to keep the model within the same range of existing human ventricular cell models [4, 5, 7, 16, 17]. The range for triangulation was 44-112 ms [31, 32]. For the diastolic and systolic [*Ca*^2+^]_*i*_ levels at 1 Hz and 0.5 Hz stimulation, the CRLP model, as occurs with other recent human ventricular AP models (GPB, ORd, TP06, TP04) [4, 5, 7, 16, 17, 33, 34], is out of the physiological range reported in the literature [35, 36]. For that reason, the physiological range was extended to ensure a feasible solution of the optimization problem. Nevertheless, the optimal solution was evaluated to confirm that either it let the model lie within physiological range or at least improved the results of the CRLP model. The physiological range for *τ*_*slow*_ was set to 70-110 s [37]. Table 1 summarizes the evaluated markers and their physiological ranges in the second column, whereas the third column indicates the lower and upper bounds used for the optimization.

**Table 1:** Electrophysiological markers evaluated in the proposed response surface approximation-based optimization.

	Physiol. Range	Selected Range	CRLP Model	This work
APD_90_ (ms)	271-366	280-310	305.6	280.9
Triangulation (ms)	44-112	44-112	78	84.6
Sys. [*Ca*^2+^]_*i*_ 1Hz (*μ*M)	1.59-2.01	0.602-2.01	0.602	0.701
Sys. [*Ca*^2+^]_*i*_ 0.5Hz (*μ*M)	0.71-1.68	0.523-1.68	0.523	0.664
Dia. [*Ca*^2+^]_*i*_ 1Hz (*μ*M)	0.20-0.33	0.097-0.33	0.097	0.100
Dia. [*Ca*^2+^]_*i*_ 0.5Hz (*μ*M)	0.14-0.32	0.091-0.32	0.091	0.096
*τ*_*slow*_ (s)	70-110	70-110	105.6	106.0
∫_*CL*_ *I*_*K*,*tot*_ ⋅ *dt* (ms ⋅ pA/pF)			-28.52	-3.9 ⋅ 10^−9^

#### 2.2.6 Sensitivity analysis

Prior to solving the minimization problem, a sensitivity analysis was performed on the CRLP model following the methodology described by Romero *et al*. [34]. The aim was to identify the conductances that most notably influenced the objective function (the integral of the total potassium current) and the constraints (electrophysiological properties) in order to define the set of decision variables for the optimization.

For the sensitivity analysis, the conductances were varied by ±30% one at a time. Fourteen conductances were considered in the sensitivity analysis, namely: *G*_*to*_, the maximal conductance of the transient outward potassium current; *G*_*Ks*_, the maximal conductance of the slowly activating potassium current; *G*_*pCa*_, the maximal conductance of the sarcolemmal calcium pump; *G*_*Kr*_, the maximal conductance of the rapidly activating potassium current; *G*_*Kp*_, the maximal conductance of the plateau potassium current; *G*_*NaK*_, the maximal activity of the Na-K pump; *G*_*ncx*_, the maximal activity of the Na-Ca exchanger; *G*_*CaL*_, the maximal conductance of the L-Type calcium current; *G*_*K*1_, the maximal conductance of the inward rectifier potassium current; *G*_*Na*_, the maximal conductance of the fast sodium current; *G*_*ClCa*_, the maximal conductance of the calcium-activated chloride current; *G*_*Na*,*Bk*_, the maximal conductance of the background sodium current; *G*_*Cl*,*Bk*_, the maximal conductance of the background chloride current; and *G*_*Ca*,*Bk*_, the maximal conductance of the background calcium current.

For each property *m* and conductance *p*, the percentage of change (*D*_*m*;*p*;*a*_) and sensitivity (*S*_*m*;*p*;*a*_) were calculated as follows [34]:

$$\begin{array}{c} D_{m;p;a}=\frac{M_{p;a}-M_{control}}{M_{control}}\cdot 100, \end{array}$$ (22)

$$\begin{array}{c} S_{m;p;a}=\frac{D_{m;p;a}-D_{m;p;-a}}{2\cdot a}\cdot 100, \end{array}$$ (23)

where *M*_*p*;*a*_ is the value of property *m* when the conductance *p* is varied by the percentage *a* and *M*_*control*_ is the value of property *m* under control conditions. As shown above, sensitivity (*S*_*m*;*p*;*a*_) is calculated as the ratio of the difference between the percentage of change (*D*_*m*;*p*;*a*_ − *D*_*m*;*p*;−*a*_) and the interval of change (2 ⋅ *a*). In particular, for this study *a* = 30%:

$$\begin{array}{c} S_{m;p;0.3}=\frac{D_{m;p;0.3}-D_{m;p;-0.3}}{2\cdot 0.3}\cdot 100=\frac{M_{p;0.3}-M_{p;-0.3}}{M_{control}}\cdot \frac{10^{5}}{6} \end{array}$$ (24)

Sensitivity was calculated for each of the electrophysiological markers described in section 2.2.4 and for the integral of the total potassium current (*I*_*K*,*tot*_) during one cardiac cycle at 1 Hz steady-state pacing.

#### 2.2.7 Model comparison

Considering the difficulty of directly comparing the absolute value of each conductance for different computational models, due to the dependence on the whole current formulation, ionic current traces calculated during a cardiac cycle at 1 Hz steady-state pacing were compared for the CRLP, TP06, GPB and ORd models. The comparisons aimed at verifying that the modified CRLP model had current densities of similar magnitude and ionic currents of similar shapes as calculated with other published models.

### 2.3 Frequency-dependence behavior

Pacing at different frequencies was simulated to assess whether the modified CRLP model retained the frequency-dependence behavior of the original CRLP model. To this end, *APD*_90_ was calculated at steady-state for CLs ranging from 250 to 5000 ms (in each case computed after pacing for 3,000 cycles).

### 2.4 Simulation of changes in potassium concentration

Hypokalemic and hyperkalemic conditions were simulated with the original and the modified CRLP models. Two types of simulations were performed to compare the models with the experimental results.

First, the effect of changing [*K*^+^]_*o*_ was assessed when the cell was not stimulated to compare with the experimental results described in [38]. Subsquently, and following the experiments performed in [18], the extracellular potassium concentration, [*K*^+^]_*o*_, was increased from 2 mM to 7 mM in 0.5 mM steps using a homogeneous 4-cm long fiber composed of epicardial cells. The physiological [*K*^+^]_*o*_ value of 5.4 mM replaced the value of 5.5 mM in the simulations [18]. The conductivity *σ*, defining the velocity of the stimulus propagation, was set to render a Conduction Velocity (CV) close to 65 cm/s under control conditions [39]. The cell capacitance was set to *C*_*m*_ = 1 *μ*F/cm^2^. For each [*K*^+^]_*o*_ value, the model was first stabilized (without stimulation) until the product of the gates *h* ⋅ *j* reached 99% of the steady-state value *h*_*ss*_ ⋅ *j*_*ss*_, which was subsequently followed by 1 Hz pacing simulation. Stimulus current pulses twice the diastolic threshold were applied at one end of the cable. The diastolic threshold was defined as the minimum amplitude required for propagating five pulses along the fiber. The model was studied by stimulating the tissue with a train of 100 basic stimulations (S1) delivered at a CL of 1,000 ms. [*K*^+^]_*i*_ was measured at the end of each stimulus at five different positions along the cable situated at distances from the origin of 1.5, 1.75, 2, 2.25 and 2.5 cm. The average value over the five locations was calculated.

### 2.5 Implementation

The CRLP model was implemented in Matlab based on the original CellML file [7]. The code used to compute the simulations and to calculate the different markers is freely available at https://github.com/ChusCarro/MatCardiacMLab/releases/tag/0.1.0.

The minimization algorithm was implemented in Matlab. For 1D tissue simulations, a semi-implicit operator-splitting scheme implemented in Fortran was used to solve the propagation [40] with a space discretization of Δ*x* = 0.1 mm and a time discretization of Δ*t* = 0.002 ms, as in [7].

All computations were performed by using the high performance computing cluster (ICTS) “NANBIOSIS”. Details about the cluster can be found at: http://www.nanbiosis.es/u27-e1-hermes-cluster.

## 3 Results

### 3.1 Sensitivity analysis

**Fig 3:**
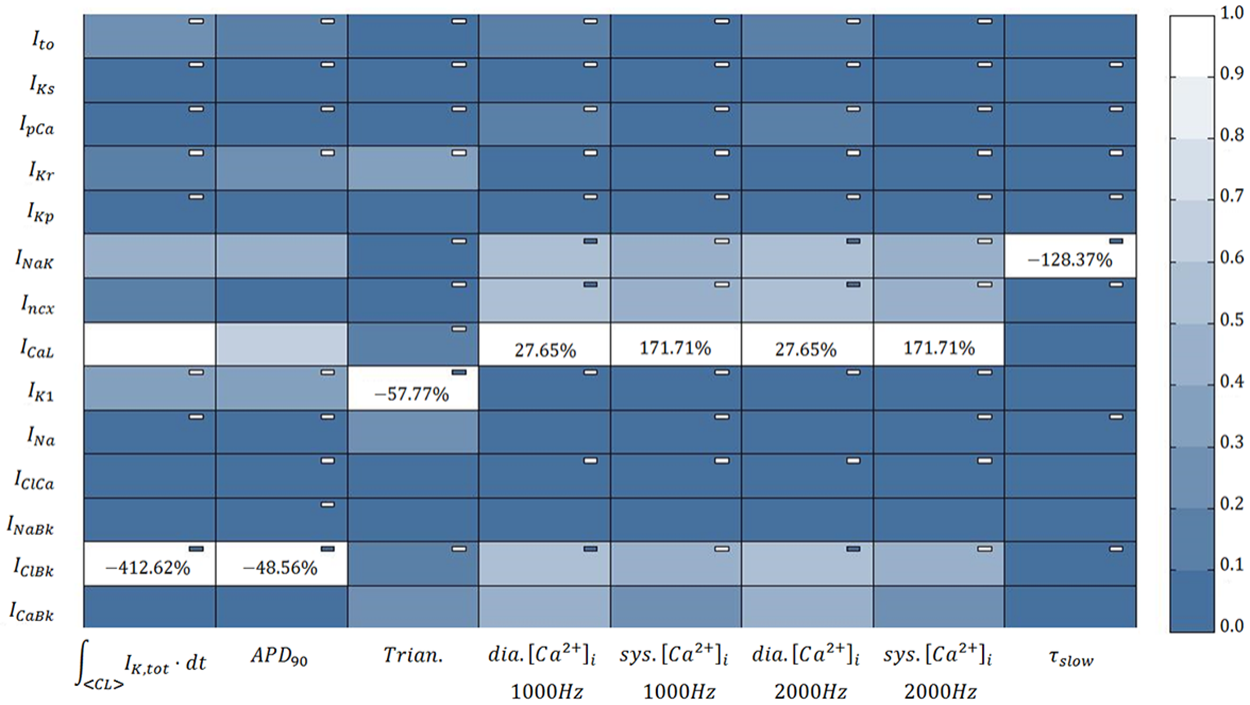
Results of the sensitivity analysis applied to the original CRLP model. The blue scale indicates relative sensitivity for each marker. White color indicates maximum relative sensitivity and dark blue color indicates that property and parameter are independent. Percentages in the white boxes indicate the absolute sensitivity of the property. A minus sign in a box indicates that marker and model parameter vary inversely. ‘Trian’ stands for Triangulation, ‘Sys’ stands for systolic and ‘Dia’ stands for diastolic.

Results of the sensitivity analysis applied to the epicardial version of the CRLP model are shown in Fig 3. The value of the integral of the total potassium current, *I*_*K*,*tot*_, in one cardiac cycle was highly dependent on the conductance of the background chloride current, *G*_*Cl*,*bk*_, and the L-type calcium current, *G*_*CaL*_ (*S*_30_ values of -412.62% and 396.83%, respectively). Other currents were also found to play an important role in regulating potassium homeostasis: *G*_*NaK*_, 199.46%; *G*_*K*1_, -152.06%; *G*_*to*_, -110.57%; *G*_*Kr*_, -81.16%; and *G*_*ncx*_ 62.72%. The currents with the largest effect on the APD were found to be the same as for the objective function, although the sensitivities were notably reduced: *G*_*Cl*,*bk*_, -48.56%; and *G*_*CaL*_, 33.41%. Triangulation was mainly modulated by: *G*_*K*1_, -57.7664%; and *G*_*Kr*_, -20.1113%. Diastolic and systolic [*Ca*^2+^]_*i*_ values were mostly influenced by *G*_*CaL*_ (27.65% and 171.71%, respectively). These markers were also influenced by changes in the following conductances: *G*_*NaK*_ (-16.08%, -78.62%), *G*_*ncx*_ (-15.74%, -74.48%), *G*_*Cl*,*bk*_ (-14.53%, -80.66%) and *G*_*Ca*,*bk*_ (11.51%, 47.46%). Regarding *τ*_*slow*_, *G*_*NaK*_ was the conductance with the largest effect on this marker (-128.37%), with the remaining conductances not playing a significant role.

Based on these results, five conductances were selected as decision variables for the minimization problem described in 2.2.3, namely: *G*_*Cl*,*bk*_, *G*_*CaL*_, *G*_*ncx*_, *G*_*K*1_, and *G*_*to*_. Of note, *G*_*NaK*_ was discarded because any modification on this conductance significantly influenced *τ*_*slow*_, with this effect not being counterbalanced by changes in any other conductance (see Fig 3).

### 3.2 Non-linear optimization

The optimization algorithm found a minimum after four iterations. Variations in the updated current conductances with respect to their initial values were: *G*_*Cl*,*bk*_, +19.34%; *G*_*CaL*_, +3.93%; *G*_*ncx*_, -33.25%; *G*_*K*1_, -14.08%; and *G*_*to*_, +22.35%.

With this new set of conductance values, and after introducing [*K*^+^]_*i*_ dynamics in the model, [*K*^+^]_*i*_ stabilized at 138 mM. The value of the electrophysiological markers are described in Table 1. All markers were within physiological ranges or closer than with the original CRLP model (last column in Table 1).

When the model was optimized while considering different initial [*K*^+^]_*i*_ values in the range 120-145 mM, results were practically coincident with those reported above for [*K*^+^]_*i*_ initialized at 138 mM. At steady-state, [*K*^+^]_*i*_ reached a value of 138 mM as well, differences in the values of the markers where below 1% and the maximum difference between optimal conductances was lower than 2.5%. In this case, the algorithm required six iterations rather than four to reach the optimal solution.

### 3.3 Model comparison

**Fig 4:**
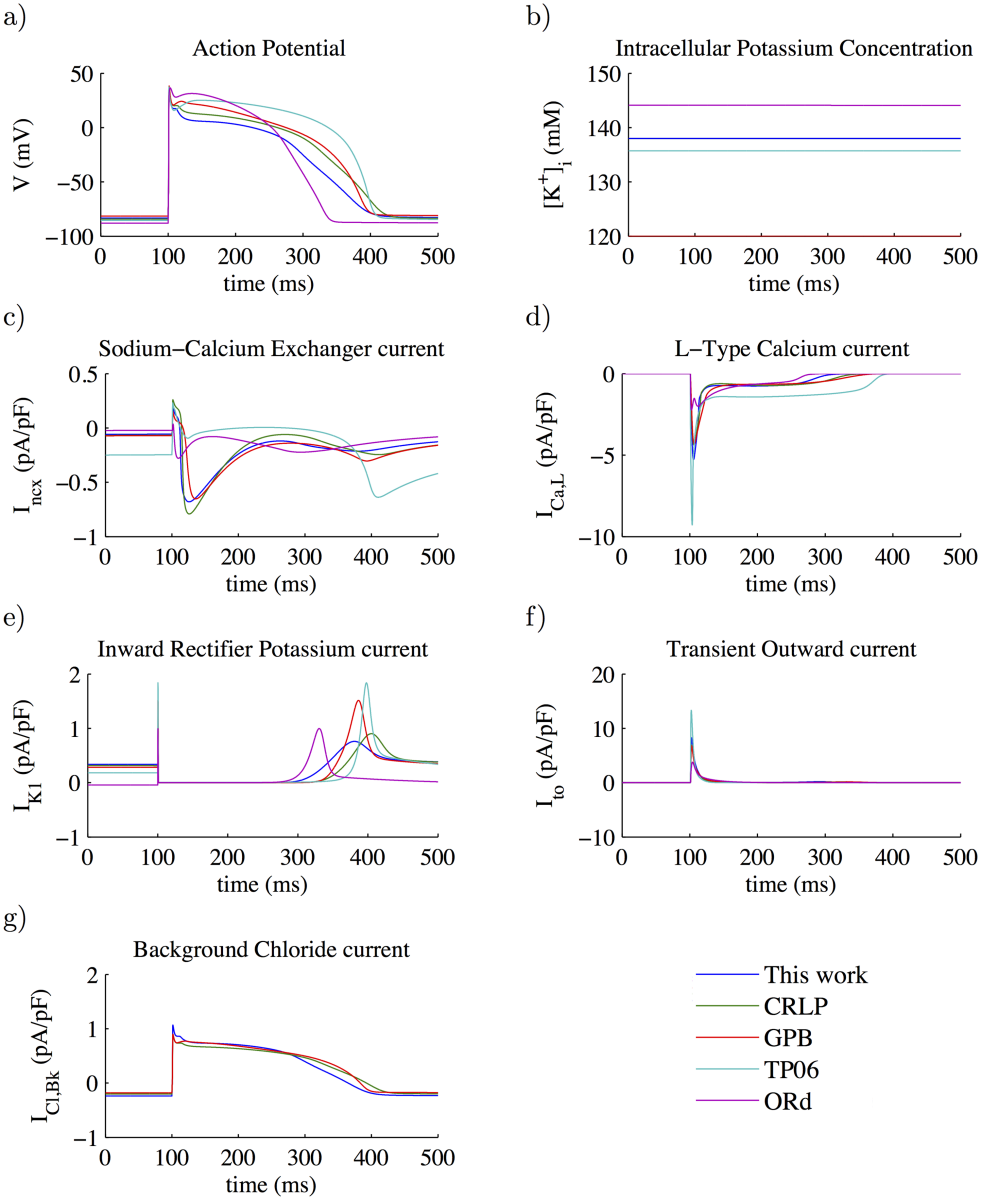
Model comparison. Blue lines represent the results of this work with inclusion of [*K*^+^]_*i*_ dynamics, green lines represent the CRLP model, red lines represent the GPB model, cyan lines represent the TP06 model and purple lines represent the ORd model. All the figures show results at steady-state (after pacing for 3,000 cycles at a CL of 1000 ms). a) Action potential; b) Intracellular potassium concentration; c) Sodium-calcium exchanger Current; d) L-type calcium current; e) Inward potassium current; f) Transient outward potassium current; and g) Background chloride Current.

**Fig 5:**
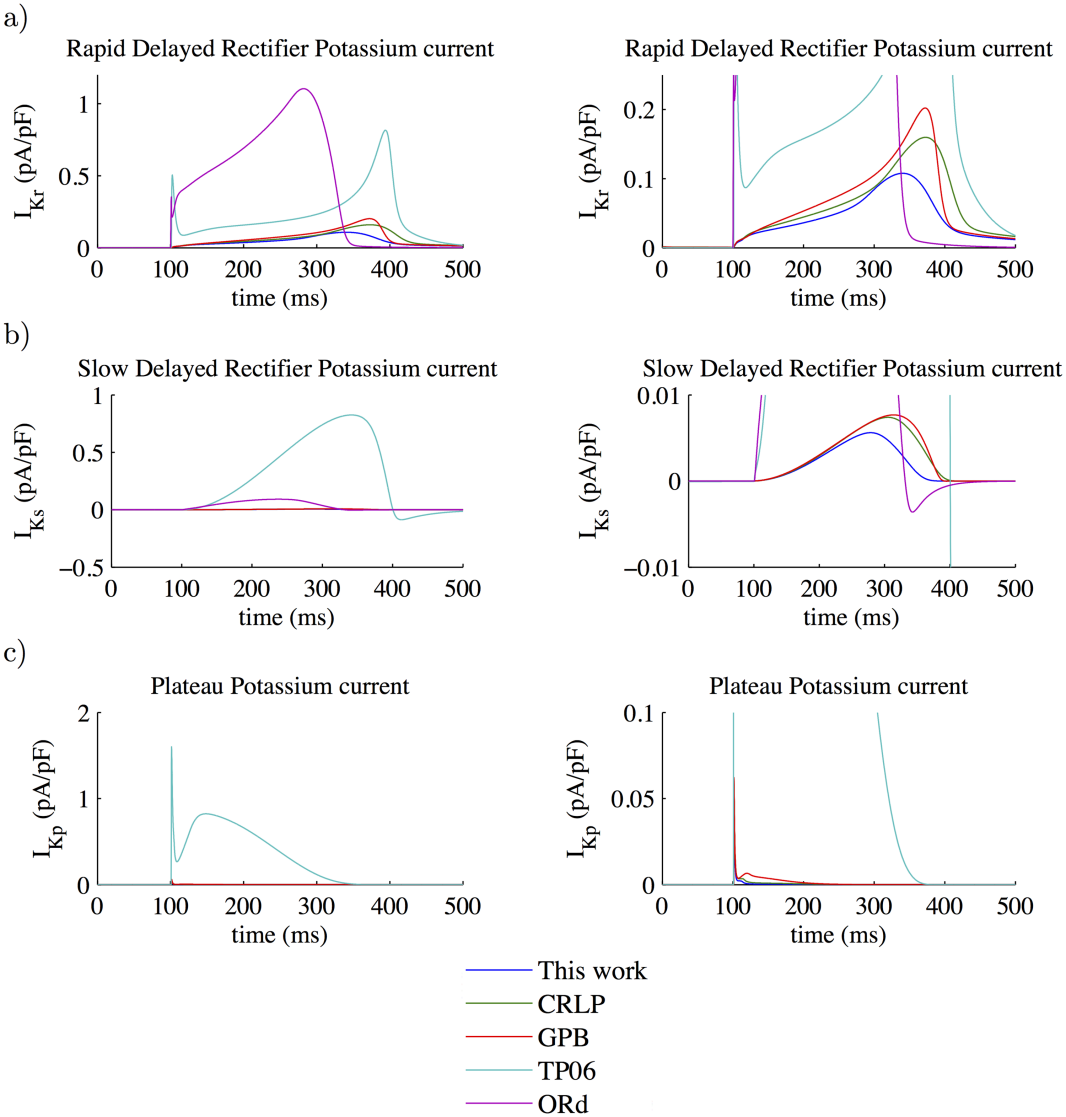
Model comparison. The colors in this figure are the same as those used in Fig 4. Results are shown at steady-state (after pacing for 3,000 cycles at a CL of 1000 ms). The graphics on the right are zoomed versions of the graphics on the left. a) Rapid delayed rectifier potassium current; b) Slow delayed rectifier potassium current; and c) Plateau potassium current.

To evaluate the impact of the new values for the estimated ionic conductances in the modified CRLP model, the AP and the associated ionic currents (*I*_*CaL*_, *I*_*K*1_, *I*_*ncx*_, *I*_*Cl*,*bk*_ and *I*_*to*_) were compared with the equivalent properties in the original CRLP model and in other human ventricular cell models. The results are shown in Fig 4. Other currents that were not considered for the optimization but that could be altered as well, like *I*_*Kr*_, *I*_*Ks*_ and *I*_*Kp*_, are shown in Fig 5.

The main differences in the AP between the original and modified CRLP models were a decrease in the membrane potential during the *plateau* phase as well as shortening of the APD. There were small differences in the peak value of some currents, but in all cases the modified CRLP model was within the range of the other models or within experimentally reported bounds. *I*_*CaL*_ peak was larger in the modified model with respect to the GPB model and the original CRLP model, but smaller than in the TP06 model. *I*_*K*1_ peak was slightly smaller in the modified model with respect to the original CRLP model, which had the smallest peak value among all ventricular models, but in any case within the range reported experimentally (0.4-1.8 pA/pF [31, 41]).

An important finding was that, while *G*_*ncx*_ and *G*_*Cl*,*bk*_ in the modified CRLP model were significantly different from those in the original CRLP model (-33.25% and +19.34% respectively), the effects in terms of ionic current traces led to both *I*_*ncx*_ and *I*_*Cl*,*bk*_ not being notably different from those in the original CRLP model. The *I*_*Kp*_ current remained as in the original model, while *I*_*Ks*_ was different but took really small values in both cases. *I*_*Kr*_ was diminished in amplitude by 32.6% and its integral during a cycle was reduced by 30% with respect to the CRLP model. The extent of APD prolongation following blockade of this current was 10% for the modified CRLP model, as compared to 14% for the original CRLP model, being both values within experimentally defined limits [42].

### 3.4 Frequency response

**Fig 6:**
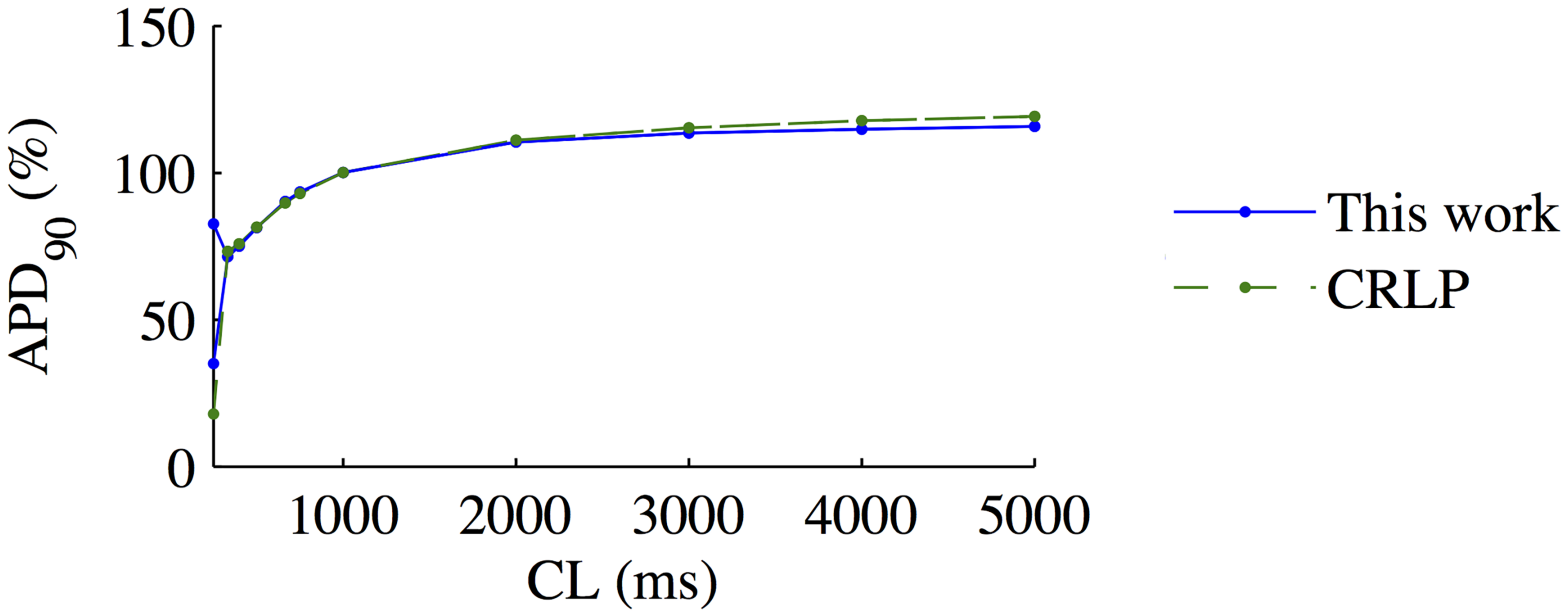
Steady-state *APD*_90_ values as a function of pacing CL.

Fig 6 shows steady-state normalized *APD*_90_ values calculated with the original and modified CRLP models. Normalization was performed with respect to the *APD*_90_ value for CL = 1000 ms. Both models presented very similar behaviors, with the only relevant difference being observed for CL = 250 ms. For this CL, the modified CRLP model showed alternans, while the original CRLP model did not.

### 3.5 Response to changes in extracellular potassium

**Fig 7:**
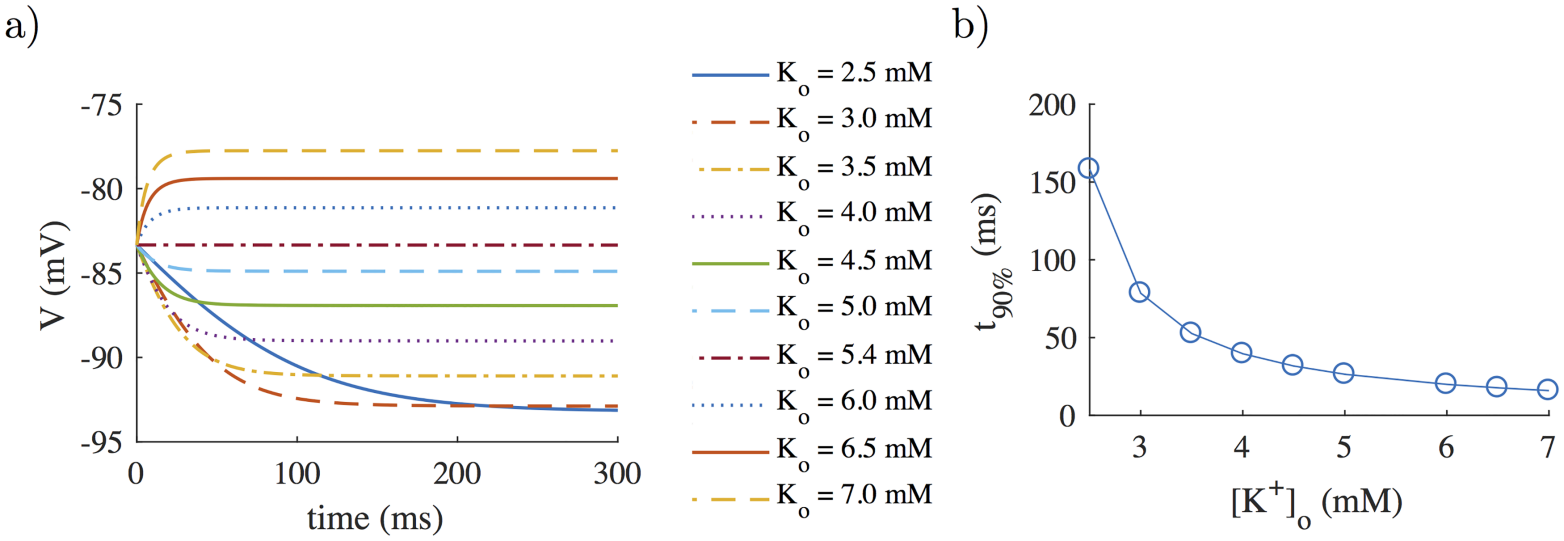
Results for an unstimulated cell simulated with the modified CRLP model following changes in [*K*^+^]_*o*_. a) Temporal evolution of membrane potential; b) Time to achieve 90% of the steady-state membrane potential (*t*_90%_).

Fig 7a shows the temporal evolution of membrane potential following changes in [*K*^+^]_*o*_ for an unstimulated cell simulated with the modified CRLP model. During the first 100 ms, membrane potential varied following the corresponding change in [*K*^+^]_*o*_, in line with the results reported in [38]. Fig 7b shows the time needed to reach 90% of the steady-state membrane potential (*t*_90%_). The value for *t*_90%_ in the simulation for the same change in [*K*^+^]_*o*_ than in [38] (from 4.4 mM to 6.6 mM) was 16.2 ms, which is somewhat faster than the experimentally reported results in [38] for rabbit (80 ms) and rat (147 ms) ventricular myocytes.

**Fig 8:**
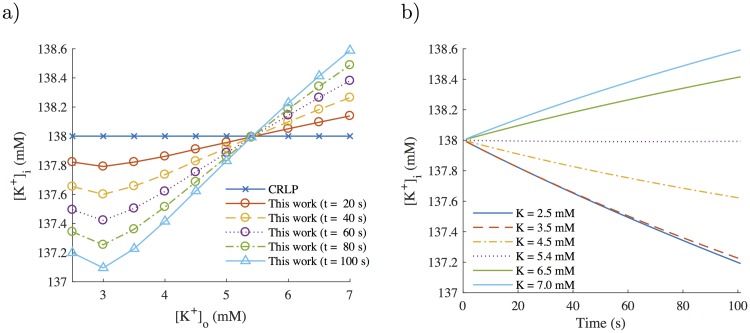
[*K*^+^]_*i*_ evolution following [*K*^+^]_*o*_ changes simulated with the modified CRLP model. a) [*K*^+^]_*i*_ dependence on [*K*^+^]_*o*_ at different time instants; b) time course of [*K*^+^]_*i*_ for different [*K*^+^]_*o*_ values.

Fig 8a shows a snapshot of [*K*^+^]_*i*_ values calculated with the modified CRLP model at different times following changes in [*K*^+^]_*o*_ as described in 2.4. Fig 8b shows the temporal evolution of [*K*^+^]_*i*_ in response to changes in [*K*^+^]_*o*_. Under control conditions, [*K*^+^]_*i*_ remained constant in time. As expected, following simulated hyperkalemia ([*K*^+^]_*o*_ > 5.4 *mM*), [*K*^+^]_*i*_ increased as [*K*^+^]_*o*_ increased, whereas for simulated hypokalemia ([*K*^+^]_*o*_ < 5.4 *mM*), [*K*^+^]_*i*_ decreased in response to [*K*^+^]_*o*_ decreases. For [*K*^+^]_*o*_ ≤ 2.0 *mM*, tissue became inexcitable. In Table 2, the steady-state value of [*K*^+^]_*i*_ in the optimized CRLP model is shown for each simulated [*K*^+^]_*o*_ value.

**Table 2:** Steady-state values of [*K*^+^]_*i*_ for different [*K*^+^]_*o*_ values as calculated with the optimized CRLP model.

[*K*^+^]_*o*_ (mM)	[*K*^+^]_*i*_ (mM)
2.5	73.5
3.0	84.8
3.5	95.8
4.0	107.2
4.5	118.3
5.0	129.3
5.4	138.0
6.0	151.4
6.5	161.6
7.0	172.5

### 3.6 Computational cost

Each iteration of the optimization algorithm involved computations associated with a number of stimulation protocols. In Table 3, a summary of the computational cost is shown for each of the protocols. In each iteration, the protocols were run twice: one for the generation of the database (243 simulations in parallel) and another one for the evaluation of the solution (6 simulations in parallel). The variance in the computation times is due to the fact that computations were run in different nodes of a high performance computing cluster, namely the one described in section 2.5. The four iterations of the optimization algorithm were computed in 23 hours and 16 minutes.

**Table 3:** Computational cost of the different protocols used for the optimization.

Protocol	Mean	STD	Max
Steady-state CL = 1000 ms	01:31:45	00:41:31	02:29:31
Steady-state CL = 2000 ms	02:35:38	01:03:47	03:41:11
APD Rate Adaptation	00:28:36	00:14:17	00:53:57

## 4 Discussion

This work proposes a mathematical framework to adjust ionic current conductances of cardiac AP models by using response surface approximation-based optimization. The framework has been applied to update a previously published human ventricular AP model, the CRLP model, by incorporating [*K*^+^]_*i*_ dynamics. Such incorporation involves readjustment of ionic current conductances to avoid drifting in intracellular potassium. While adjusting for current conductances, a number of electrophysiological properties are required to remain within available experimental ranges by properly defining constraints to the optimization problem that is being solved. The proposed framework can be easily extended to solve other problems frequently arising when developing cardiac computational models, for instance related to the update of existing models to reproduce the response to pharmacological ion channel inhibitions on top of baseline conditions. In the following subsections, the main aspects of the proposed methodology and the results obtained in this work are discussed.

### 4.1 Response surface optimization

The use of response surfaces to minimize an objective function subject to a number of constraints, as proposed in this study, allows reaching a solution to the proposed problem in a small number of iterations, thus reducing the computational cost with respect to other previously published approaches. In [15], Rodriguez *et al*. proposed an algorithm that uses augmented Lagrangian techniques and relies on successively building RSA of the augmented Lagrangian to solve a minimization problem like that defined by Eqs 1–4. In this study, the trust region ratio was used to independently validate the RSA for the objective function and the constraints instead of considering the augmented Lagrangian of the problem. Our proposal led to a reduction in the number of times the database was generated and, thus, simplified the implementation. The model that is optimized in the present work is much more complex than those used in [15], but the number of times the database was generated was smaller than in all the cases described in [15].

Also, differently to other optimization algorithms based on e.g. Genetic Algorithms [12, 14], the response surface approach proposed in this study guarantees the convergence to a local minimum of the problem when the response surfaces are built such that they match the objective function up to the first order around the point where the approximation is built. In addition, our response surface approach allows identifying a potential minimum anywhere within the trust region, not necessarily contained in the database.

Finally, in this work the proposed optimization algorithm was used to solve a problem where a new characteristic was added to a human ventricular cell model, the CRLP model, while a number of electrophysiological markers were guaranteed to remain within physiological range or closer to it than with the original CRLP model. In other studies, like [43], the aim of the analysis is similar, but, as opposed to the present study, the objective function is defined to include multiple electrophysiological properties, for some of which a physiological range may be available while for others it may not, rather than considering the electrophsyiological properties as elements to define the constraints imposed in the optimization. In any case, the same response surface approximation-based optimization algorithm employed in the present study could be applied to the problem defined in [43].

### 4.2 Database generation

The size of the database depends on the selected sampling technique. In this study a three-level full factorial design was used for sampling, which involved 3^*N*^ sample points, with *N* the number of variables to be adjusted (five in this study). However, the proposed optimization framework is independent of the sampling strategy or the size of the database as long as the database provides enough information to fit the response surface. For instance, increasing the factorial number from 3 to 5 (the database size increased from 3^5^ = 243 to 5^5^ = 3125 sample points) only reduced the total number of iterations from 4 to 3, without changing the identified optimum. However, the computational cost increased tenfold.

The advantage of using factorial designs is that they provide unbiased information for parameter identification with polynomial RSAs since they provide optimal space filling [19]. However, other sampling strategies are also possible. Monte Carlo simulations represent a very flexible sampling strategy where the optimal number of sampling points can be defined in terms of the type of RSA to be used. In this regard, Monte Carlo simulations were used in [44] to perform sensitivity analysis in electrophysiological models by means of linear multivariate regression. In addition to generating the required data for model fitting, Monte Carlo simulations were also used to perform population-based simulation studies, as in Wamsley *et al*. [45].

In this work, the full factorial technique was implemented in order to guarantee that the algorithm was not limited by the sampling technique since the generated database is sufficient to build a second order response surface. However, as mentioned above, other techniques could be tested to reduce the computational cost when a larger number of current conductances are involved or when large parallel computing capacity is not available.

### 4.3 Computational cost

The major benefit of the methodology proposed in this study in terms of computational cost is the high degree of parallelization. It should be noted that the global computation time depends mainly on two factors: the employed cell model and the stimulation protocols that are performed. As compared to the CRLP or GPB models, which take long computation times, the computational time for a model like TP06 could be reduced to just a few hours. For the computations of this study a high performance computing cluster was used. The number of simulations involved per iteration makes it possible to use other types of infrastructures such as GPUs.

Nevertheless, the computation times associated with the presented response surface approximation-based methodology are short if compared with those for the same optimization solved by using a genetic algorithm, which could require around 100 generations and would cost more than 350 hours, even if all individuals in the population were simulated in parallel. In [12], 500 generations were needed to fit the model, which would still involve longer computation times.

### 4.4 Definition of the optimization problem

In this work, the integral of the total potassium current (*I*_*K*,*tot*_) during one cardiac cycle was selected as the objective function for minimization. This selection was made to maintain [*K*^+^]_*i*_ homeostasis after incorporation of [*K*^+^]_*i*_ dynamics into the CRLP human ventricular model. Optimal current conductances were identified, with additional constraints imposed to guarantee that fundamental electrophysiological properties took values within experimental limits. For some of those properties the physiological range reported in experimental studies of the literature is extremely large. To avoid a solution for the modified CRLP model that, although in physiological range, was very far from the average experimental behavior, the range of allowed APD values was somewhat restricted, as described in 2.2.5.

### 4.5 Model comparison

The adjusted CRLP model developed in this study was compared with previous human ventricular AP models published in the literature, namely the CRLP, GPB, TP06 and ORd models. This comparison was made in terms of AP traces, [*K*^+^]_*i*_ concentrations and *I*_*CaL*_, *I*_*K*1_, *I*_*ncx*_, *I*_*Cl*,*bk*_ and *I*_*to*_ currents. In practically all cases, the results obtained for the adjusted CRLP model were along the lines of those obtained with the other models. The only current that was found to be smaller for the modified CRLP model as compared to the other models, including the original CRLP model, was *I*_*Kr*_. This reduced current led to shorter APD prolongation following *I*_*Kr*_ blockade, but still within experimentally reported limits. Specifically, in [42] an average APD prolongation of 56.3% with a standard deviation of 34.6% was reported. The difference between the mean experimental prolongation and that obtained for the modified CRLP model was within twice the experimental standard deviation.

One of the differences between the GPB / CRLP models and the other human ventricular models is the inclusion of the *I*_*Cl*,*bk*_ current. This current was expected to have a minor role in modulating electrophysiological properties, but, as reported in [33], APD showed the highest sensitivity (-86%) to changes in the maximal ionic conductance of the *I*_*Cl*,*bk*_ current. In the CRLP model, this sensitivity was reduced (-46%) but it was still large. As there is similarity between the sensitivities of the APD and the integral of the total potassium current to variations in ionic conductances (see Fig 3), the *I*_*Cl*,*bk*_ current ended up having a very important indirect role in modulating potassium balance.

As shown in the presented results, major differences between the modified and the original CRLP models include APD shortening and decrease in the membrane potential during the *plateau* phase. Regarding the latter, experimental results [46] show that the plateau voltage takes values around 18 mV, while in the modified CRLP model the plateau voltage is 8 mV. Differences in *I*_*ncx*_ and *I*_*Cl*,*bk*_, even if not large, could still underlie those major deviations of the modified CRLP model with respect to the original one. Future studies could include the membrane potential during the *plateau* phase as an electrophysiological marker to be included in the optimization algorithm for model refinement. This could definitely have an impact on balancing the currents involved in this phase of the AP.

Our results demonstrate that with the proposed methodology it was possible to successfully update the CRLP model by incorporating [*K*^+^]_*i*_ dynamics while keeping the model’s ability to reproduce risk-related markers for investigation of cardiac arrhythmias. As described in Table 1, the evaluated risk markers remain within experimental limits or are closer to their physiological values than with the original CRLP model.

### 4.6 Response to potassium concentration changes

While for some investigations the assumption of constant [*K*^+^]_*i*_ may be valid, there are many situations where [*K*^+^]_*i*_ varies, for instance following changes in the frequency of stimulation or in [*K*^+^]_*o*_ values. As shown in 2.2.2, the assumption of constant [*K*^+^]_*i*_ may also hide an imbalance between potassium currents and [*K*^+^]_*i*_, as the latter is not allowed to change.

Simulation results obtained when varying the level of [*K*^+^]_*o*_ allowed confirming that both the incorporation of [*K*^+^]_*i*_ dynamics and the use of the optimization algorithm to readjust the ionic conductances improved the CRLP model for simulation of abnormal conditions (see section 3.5). Although there are no experimental measurements of [*K*^+^]_*i*_, the model can be assessed by using measurements that indirectly validate its behavior. On the one hand, the model is able to reproduce behaviors similar to those of the experimental results presented in [38]. On the other hand, the inclusion of [*K*^+^]_*i*_ dynamics make it possible to evaluate variations in [*K*^+^]_*i*_ in response to [*K*^+^]_*o*_ changes like those reported in hemodialysis scenarios [18]. In this latter case, simulated [*K*^+^]_*i*_ variations were in the expected direction as to compensate for the effects of changing [*K*^+^]_*o*_.

When [*K*^+^]_*o*_ increases, there is an increase in the Nernst potential for potassium and, thus, a reduction in the driving force of potassium, which diminishes the amount of potassium leaving the cell. Additionally, an increase in [*K*^+^]_*o*_ alters the current through the sodium-potassium pump. Both effects contribute to increase [*K*^+^]_*i*_, which leads to a reduction in the Nernst potential for potassium, thus returning the cell to an equilibrium state.

## 5 Conclusions

The optimization framework proposed in this study allows estimating ionic current conductances of AP models at an affordable computational cost while guaranteeing physiologically plausible values of selected electrophysiological properties. This framework has been applied to introduce [*K*^+^]_*i*_ dynamics into an existing human ventricular AP model, namely the CRLP model. Solving the formulated optimization problem avoids the imbalance generated in the CRLP model after introducing [*K*^+^]_*i*_ dynamics, without deteriorating the performance of the model in terms of representation of electrophysiological behavior and arrhythmic risk-related properties. The updated CRLP model built in this study is thus suitable for investigation of cardiac arrhythmias, particularly under conditions involving large changes in [*K*^+^]_*i*_.
